# Feasibility Assessments Using Unmanned Aerial Vehicle Technology in Heritage Buildings: Rehabilitation-Restoration, Spatial Analysis and Tourism Potential Analysis

**DOI:** 10.3390/s20072054

**Published:** 2020-04-06

**Authors:** Paul Sestras, Sanda Roșca, Ștefan Bilașco, Sanda Naș, Stefan M. Buru, Leontina Kovacs, Velibor Spalević, Adriana F. Sestras

**Affiliations:** 1 Faculty of Civil Engineering, Technical University of Cluj-Napoca, 400020 Cluj-Napoca, Romania; sanda.nas@mtc.utcluj.ro (S.N.); marius.buru@mecon.utcluj.ro (S.M.B.); 2 Faculty of Geography, Babes-Bolyai University, 400006 Cluj-Napoca, Romania; sanda.rosca@ubbcluj.ro (S.R.);; 3 Cluj-Napoca Subsidiary Geography Section, Romanian Academy, 400015 Cluj-Napoca, Romania; 4 Cadastre and Land Registration Office, 400436 Cluj-Napoca, Romania; leontina.kovacs@ancpi.ro; 5 Faculty of Horticulture, University of Agricultural Sciences and Veterinary Medicine of Cluj-Napoca, 400372 Cluj-Napoca, Romania; 6 Geography Department, Faculty of Philosophy, University of Montenegro, 81400 Niksic, Montenegro; velibor.spalevic@ucg.ac.me

**Keywords:** 3D model, accessibility study, GIS analysis, monumental heritage, point cloud, remote sensing, structure from motion (SfM), topographical survey

## Abstract

The Transylvanian region of Romania is a place of rich history since ancient times, where the original natural environment around architectural heritage sites or buildings has not been severely altered by urban development. Unfortunately, many such places are left by the authorities to degrade or totally collapse for lack of funds, vision or initiatives. The current paper addresses the potential of Unmanned Aerial Vehicles (UAVs) in the assessment of a viable and feasible prospect of restoration on a 19th century mansion that belonged to a nobiliary family. UAV use is rising in many industries and has become very popular in the last decade, but for survey engineering and related domains they represent a quantum leap in technology. Integrating UAV-acquired data and structure from motion software, has enabled modern techniques to obtain useful metrics from the field, accurate photorealistic 3D models for visual inspection, structural damage analyses, architectural rehabilitation-restoration, conservation and spatial analysis of the surrounding area. In this work a socio-cultural planning and design process is explored and presented to improve the local community and inclusion in a tourist circuit based on the regional potential, as well as an evaluation of accessibility derived from a vector-raster database that highlights the central position of the cultural heritage in regards to the axis of circulation between the important metropolitan areas and the local tourist attractions. This established workflow of modern topographic and construction measurements is fully integrable into the architectural process, building information modelling, heritage conservation and reconstruction.

## 1. Introduction

In the field of architectural documentation and cultural heritage as well as numerous other disciplines and industries, Unmanned Aerial Vehicle (UAV) imaging and data acquisition has developed rapidly, facilitating compelling advancements in documentation and analysis throughout three-dimensional (3D) modelling and spatial analysis used for preservation and management of cultural heritage sites [1,2]. Drone-based surveys constitute a very important instrument of knowledge and information preliminary to any work of restoration, rehabilitation or conservation [3].

With the emergence of different innovative systems such as surveys based on UAVs equipped with a digital camera, Terrestrial Laser Scanning (TLS), total station and Global Navigation Satellite System (GNSS), 3D reconstruction is easier than before. The accessible instruments and techniques create high-quality results for 3D reconstruction and have become frequent practice for recording historical or cultural heritage objects. Photogrammetry applications have a long history of successful achievements in 3D recording and architectural documentation [4,5]. Photogrammetry techniques based on drones equipped with a digital camera have become some of the most promising and practiced techniques in last years. Factors explaining the popularity of UAV-based surveys are the efficiency of acquisition data on heritage sites of all sizes, the relatively cheap operational expenses, the rapidly captured high-resolution images and overall maneuverability in all terrain conditions [6,7,8]. The implementation of UAVs opens various new interdisciplinary applications and research directions and has become progressively common due to the considerable potential in terms of accuracy, cost and abilities [7,9,10].

Following the evolution in image capturing and processing, UAVs have become a reliable alternative in the cultural heritage domain, architecture, land monitoring and geophysical surveys. With the particularity to perform in inaccessible or high-risk situations without any danger for the user or researcher and the ability to reach normally inaccessible places, drone solutions have become one of the most popular platforms in the cultural heritage sector. Their applications are mainly focused on observation, monitoring, mapping, 3D modelling and 3D reconstruction as well as other related implementations in archaeometry, remote sensing, environment, geophysics etc. Such implementations enable modern techniques to obtain digital maps, georeferenced orthophotos that provide useful metrics from the field, essential digital elevation models (DEM) and digital surface models (DSM) further used in spatial analysis and Geographic Information System (GIS) applications [11,12,13].

Depending on the objective and desired result, modern and alternative survey solutions such as UAV systems can be superior to a traditional survey due to the capability of producing high definition images, measurements, transmit and store data. Even though the precision obtained from a UAV flight depends on the atmospheric and climatic conditions, surface and vegetation, camera and sensors used, it cannot compete with the millimetric precision of a total station or a terrain laser scanner. The main advantage is the multitude of points obtained, flexibility and efficiency, offering a surface representation that would be impossible with other instruments. UAV-obtained data combined with specialized software for generating accurate and complex 3D models, consists of a tremendous number of point clouds that deliver formidable outputs and results. The generated deliverables consist of: digital elevation models, contour lines, longitudinal and transversal profiles, 3D models of buildings, assessment of heritage conservation through 3D model reconstruction and analysis of damage, dense cloud similar to the ones obtained by a terrain laser scanner, as well as different volumetric and surface calculations [14,15]. Such advantages can also benefit the social-economic spectrum, improvements in productivity, real-time data collection, cost-effective surveys of entire construction sites and architectural process and supervised assistance on site. Drones have become a great platform to carry specialized sensor and performance cameras, replacing expensive, inaccurate in difficult circumstances and time-consuming manual data collection. Incorporating the acquired data into software such as Agisoft Metashape or other structure from motion software, Global Mapper and Geographical Information Systems (GIS) will enable a wide variety of spatial analysis that can increase the volume, accuracy and speed of producing topographic maps, land-related services, construction surveying, volumetric calculations, surface areas and other metrics, hazard and risk assessment maps, 3D models of existing buildings in order for conservation and restoration of cultural heritage, buildings inspections, monitoring and other operations for a better decision-making process [16,17].

In this case study, a heritage conservation assessment was achieved through the 3D model reconstruction of a building in an advanced state of degradation. By using low-altitude UAV flights, data processing and remote sensing technology, the 3D scans and models obtained are a major contribution in numerous domains. Until recent times, 3D laser scanners were used in similar projects. Being ground stations, they require a tripod, multiple positions, long periods of observations and are difficult to maneuver in harsh conditions. Also, being limited to the ground, TLS is unable to scan rooftops, tall structures, rough terrain, tree canopy and vegetation, thus it is strongly recommended that any laser scan project be accompanied with a flight. With the help of UAVs, 3D models of buildings and terrain can easily survey the building shape, color, texture, style, damage, structural features, and provide damage analysis. They possess invaluable importance in the architectural and archaeological field, in the process of heritage conservation, rehabilitation-restoration from the early feasibility stage to the final project [18,19].

The current paper addresses the potential of UAVs in order to analyze, inspect and interpret, conserve and manage cultural heritage data through a case study of a 19th century mansion situated in Sălaj County (Transylvania Region, Romania) that belonged to the Hatfaludy family. It also presents a procedure for processing the obtained UAV data in order to create an accurate 3D model, digital elevation and surface models, photo-realistic outputs for digital reconstruction for visual inspections, structural damage analyses, architectural rehabilitation-restoration and spatial analyses of the surrounding area. Besides the previously mentioned desideratum, the results obtained from the case study are accompanied by a touristic potential analysis to further evaluate the current cultural heritage documentation, socio-cultural and economic aspect by proposing a future rehabilitation, restoration and transformation of the mansion as a pension and restaurant based on the region’s potential. A second spatial analysis is further established, involving the accessibility from a temporary point of view that takes into account distance ratio in kilometers, time (minutes) and the average speeds of movement on different categories of road (expressways, national road, county road) and pedestrian access. The favorable position of cultural heritage case study in terms of central location in regards to the axis of circulation between the important metropolitan areas (Cluj-Napoca and Zalau) and the adjacent numerous local tourist attractions, highlight the feasibility of a large-scale investment project. 

The established interdisciplinary workflow that combines modern survey engineering, geomatics techniques, tourism output and accessibility assessment represents a novelty approach that can be fully integrated in numerous disadvantaged areas in order to regenerate the economic and social aspects, as well as to raise awareness of unexplored regions and cultural heritage sites with high potential of development. The proposed approach can serve as a guideline and methodology to both the private sector in search of potential investments and expansion, as well as the public sector by providing an approachable procedure for government institutions, local authorities and heritage conservation staff in order to identify viable and feasible buildings and sites worth development.

## 2. Materials and Methods

### 2.1. Study Area

**Figure 1:**
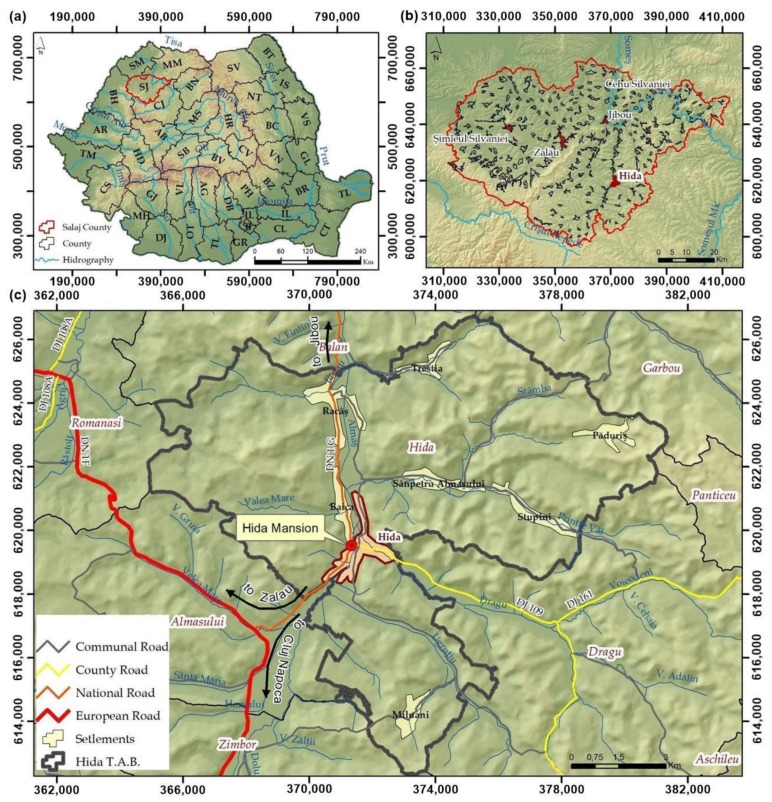
The geographic location of the study area (**a**) Romania map; (**b**) Sălaj County; (**c**) Hida administrative territorial unit.

Sălaj County is situated in the Transylvania region, in the northwest of Romania (Figure 1), at the cross between the Eastern Carpathians and the Apuseni Mountains. Sălaj County is known from ancient times as Sylvania, meaning Forest Country. From a geographical point of view, Sălaj County is an area of hills and depressions, and has a moderate continental climate. The history of the region is a rich one, being an area of interest due to the resources, location and relief from the Roman period, so that Dacian and Roman vestiges are widespread in almost the entire county. Currently, with a number of inhabitants below 300,000, Salaj County occupies the 3rd place in the counties with the smallest number of inhabitants at the national level. The economy of Sălaj county is characterized as an industrial-agrarian economy, and the tourism is underdeveloped due to the lack of infrastructure for tourist access or cultural objectives, but also to the poor promotion of the natural tourism potential of the area. The commune of Hida is located in the south-east part of Sălaj county, in the Depression of Almaş, the geographical coordinates of Hida being 47°4′2″ N 23°18′48″ E. The economic potential of the commune is high in comparison with other communes in the county, and also the tourism due to the location and tourist objectives in the area.

### 2.2. Historic Background

**Figure 2:**
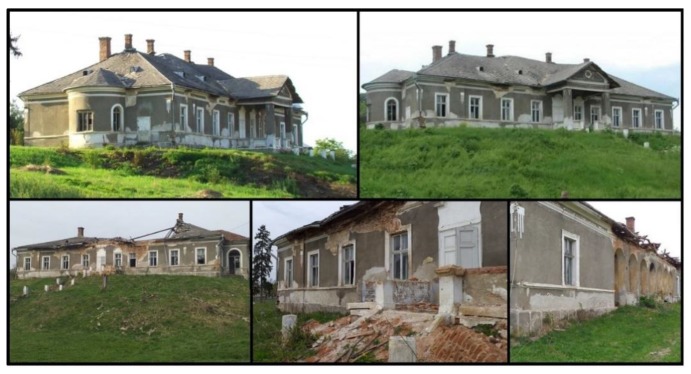
Image from a few years ago (**top**); current state of the mansion (**bottom**).

The Hatfaludy Mansion in Hida was built in the 19th century by the Hatfaludy family, a noble Hungarian family, for their son, István. The architecture style was eclecticism. The mansion was inhabited by the family until its nationalization during the communist regime. After nationalization during the communist regime, the Hatfaludy Mansion became the headquarters of the State Agricultural Enterprise in the region, being subjected to continuous destruction and degradation. In 2001, the heirs of the Hatfaludy family reclaimed the mansion, later selling it to a private enterprise. Currently, the mansion is in an advanced state of degradation, the roof being almost completely destroyed, and the interior heavily damaged and in danger of total loss (Figure 2).

In their prime, the noble residences were true architectural jewelry that hid hundreds of years of history. With few exceptions, the castles and mansions of Sălaj County are in different stages of degradation. Such monuments could increase the tourist attractiveness of the county, but sadly most of the castles and mansions in the region were left to degrade by the authorities, for lack of funds and initiatives, but also for the failure of those who bought or owned them. Such a project of feasibility assessment, restoration with the help of modern UAV techniques of data collection and post-processing, spatial analysis and accessibility study, followed by a sustainable vision of the cultural heritage can restore to the tourist circuit the forgotten residences of the former elites. Castles and mansions in Salaj county have great tourist potential, which justifies a rehabilitation-restoration project and their inclusion in a tourist circuit.

### 2.3. Tourism Potential Analysis

**Figure 3:**
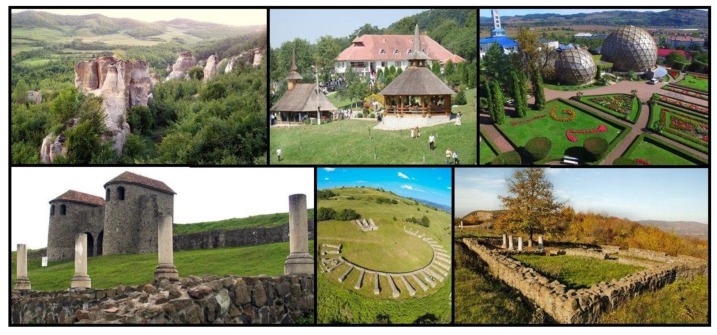
Local tourist attractions; Dragons’ Garden (**top left**), Strâmba Monastery (**top center**), Jibou Botanical Garden (**top right**), Roman city of Porolissum (**bottom**).

Sustainable development of tourism based on the natural and anthropic potential of Sălaj County is a primary objective that should be pursued by local authorities, aiming to attract funds for investments in anthropic objectives and in developing the access infrastructure. The enhancement of the natural tourism resources and the use of the existing tourist infrastructure are the basis for obtaining goods and services offered to the tourists. According to the specialized studies, the number of tourists visiting tourist objectives in Sălaj county has been increasing in recent times, creating the opportunity for tourist circulation due to the possibilities of capitalizing on attractions close to Hida Village and Hatfaludy Mansion such as: Jibou Botanical Garden, Dragons’ Garden, which is a protected natural reserve unique in the world and impressive through its aspect and the complexity of the rocks, Strâmba Monastery, one of the old orthodox monasteries of Transylvania founded in the 15th century, and Porolissum, an ancient Roman city and military camp, one of the largest and best-preserved archaeological sites in Transylvania (Figure 3).

The assessment of the tourism potential of a territory (at the level of administrative territorial unit, county, geographic region, country etc.) is carried out taking into account the natural tourist resources (natural framework, existence of natural therapeutic factors, proximity of natural protected areas, etc.) of anthropic tourist resources (which includes existence of historical monuments, museums and public collections, objects of folk art and tradition, etc.). The existing infrastructure is also evaluated taking into account the specific tourist infrastructure (the tourist accommodation functions, the existence of conference rooms, exhibition centers, etc.) and the technical infrastructure (accessibility to the major transport infrastructure, the public infrastructure and the telecommunications infrastructure). Taking into account all these factors, Sălaj County is one of the few counties in Romania that has overwhelming expansion potential. The modernization of the old mansions, especially Hatfaludy Mansion in Hida Village and their inclusion in various tourist circuits will lead to attracting more tourists and implicitly to the development of the area.

### 2.4. Methodological Approach

**Figure 4:**
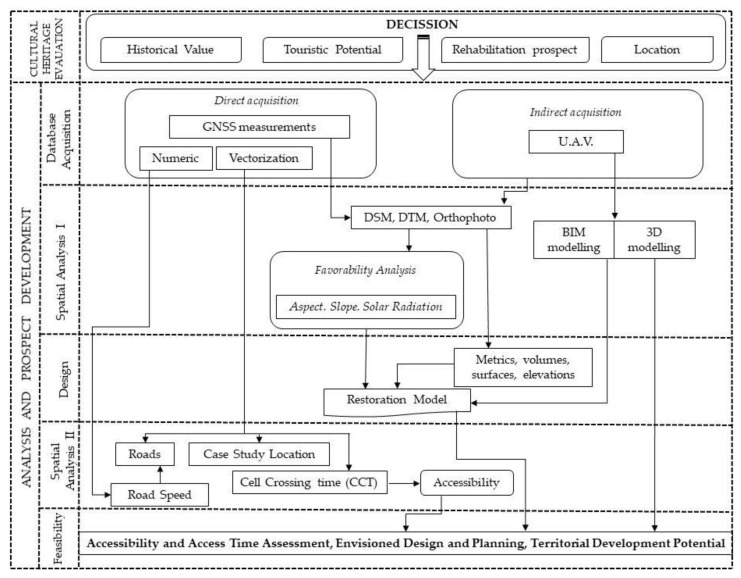
Methodological flowchart.

The methodological structure pursued to develop the presented study is in accordance with the general line of technical studies with implications for tourism and the evaluation of the tourism potential of heritage buildings and sites through the techniques and the basis of geo-informational software. Thus, in the present study, the research direction was divided in two main stages of analysis: that of evaluating the cultural heritage in order of making correct decisions regarding the interventions for future rehabilitation, landscaping and inclusion in the touristic circuit of heritage attractions (decision taken as a result of the historical value and background; the study of the tourist potential and the analysis of the location index); the second main stage, represents the analysis and prospect development that incorporates in its structure several sub-stages with technical characteristics based on acquisition of databases, 3D modelling and spatial analysis in the GIS environment (Figure 4).

The technical stage of implementing the GIS software and models for spatial analysis based on directly acquired databases (GNSS measurements, vectorization, alphanumeric data) and indirectly through the acquisition of the primary databases used in the process of 3D modeling of the heritage objective (images captured using UAV) is the basic and compulsory stage in a spatial analysis and GIS study. Thus, a model of spatial analysis based on the UAV technique was derived, whose main purpose was to carry out a favorable analysis from the point of view of the location of the manor in relation to the constraints imposed by the relief. Based on the analysis of the point cloud resulting from the processing of UAV images, raster databases have been derived (DEM, aspect, slope), databases that clearly highlight the support role that the relief has regarding the location and orientation of the analyzed construction.

Also, in the first stage of the spatial analysis the 3D model was derived and subsequently integrated in a BIM platform, allowing for a better and faster analysis of the existing situation based on the direct acquisition of volumes, surfaces, elevations and different metrics useful in the design and planning stage of restoration.

The second model of spatial analysis represents the accessibility from a temporary point of view based on the average speeds of movement on different categories of road and pedestrian access. This highlights the need for a viable rehabilitation and redevelopment due to the central position of Hatfaludy Mansion in regards to tourist circulation axes that transit the region and the potential of developing the area as a result of the introduction in a tourist circuit. The last stage is the feasibility study that involves the use of the databases obtained by developing the spatial analysis models and the analysis of the obtained 3D model, envisioned designs and utility proposed for the rehabilitation of the cultural heritage. 

### 2.5. Data Aquisition

**Figure 5:**
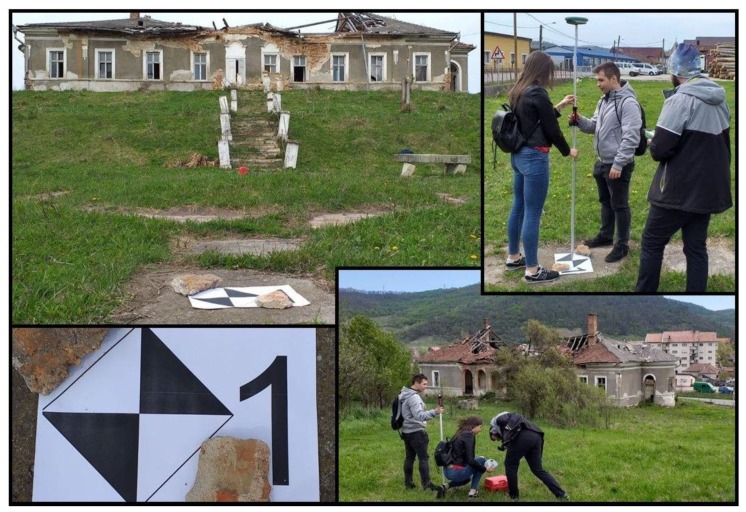
GCPs and CPs GNSS RTK measurement.

The topographic survey is one of the most well-established and accurate methods, but requires lengthy survey times, high logistic difficulties and was eventually replaced with modern methods that provides high resolution measurements directly in 3D, such as laser scanner or photogrammetry. Topography and single point measurements methods for ground control points (GCPs) was a crucial step necessary in the photogrammetry process (Figure 5). 

Digital photogrammetry techniques acquire two-dimensional images that can be further used after elaborate mathematical processing to derive 3D information. Through complex and precise formulations, processing power and software, based on projective or perspective geometry it converts the data extracted from the images into three-dimensional metric coordinates and colors. Medium-cost UAVs and structure from motion algorithms and software can be used to obtain impressive outputs with reported positive metric results, such as with centimeter-level accuracy. Due to the required case study, difficult terrain conditions associated with surveying the presented architectural heritage building, this paper assesses the reliability of UAV method combined with abundant GCPs to ensure metric accuracy and the viability as an affordable and efficient alternative to the use of TLS or traditional survey [20,21].

For accurate planimetric and altimetric positioning of ground control points (GCPs), a Viva GS08 Global Navigation Satellite System was used in Real Time Kinematics (RTK) mode. This topographic and geodetic instrument measures the coordinates of the center of the GCPs with a horizontal precision of 0.014 m to 0.030 m and vertical precision of 0.030 m to 0.050 m at each point. To ensure this level of accuracy, online RTK corrections were used provided by the Romanian Position Determination System (ROMPOS), a service provided by the National Agency for Cadaster and Real Estate Advertising. For RTK corrections, a Virtual Reference Station (VRS) was used, calculated among the closest the national permanent GNSS reference stations.

### 2.6. Flight Metrics and Error Estimates

The heritage survey was obtained using aerial images acquired with a UAV-based camera. The drone used was a DJI Mavic Pro equipped with a 4K camera, manufactured by Da-Jiang Innovations Science and Technology Co., which is a semi-professional medium-cost unit. The drone is equipped with an active stabilizing camera cradle head in order to compensate for the UAV vibrations and the wind-induced tilt, and the 4K camera ensures sharp images.

The flight mission was programmed using a specialized open source application so that a reasonable degree of overlap is ensured (Table 1). There were two stages of data acquisition, the first one was an automated flight plan to obtain a camera network that created nadir images to further georeference and postprocess to obtain orthophoto, digital mapping, DEM and DSM used for flight metrics and spatial analysis in GIS. The second stage was performed using a manual flight in order to acquire images from all possible angles to ensure the creation of an accurate 3D model of the heritage building [2,20,22].

A total of nine GCPs distributed over the study area were used as ground reference during the georeferencing process. Moreover, GCPs must be spread across the heritage and terrain area of the flight zone and is necessary to cover both high and low elevations. To assess the accuracy and precision of the final position in the created geometric model, the ground control points coordinates were located in the orthophoto images using the Agisoft Metashape software. The deviations were calculated based on the cartesian directions northing, easting and elevation. From the nine total GCPs used, five of them with the best distribution were used as GCPs in the georeferencing process, and the remaining four were used as check points (CPs). The results highlight that the calculated RMSE values were 0.034 m in the horizontal direction and 0.041 m in the vertical direction, which are acceptable results for georeferencing and sufficient for the majority of engineering projects (Table 2 and Table 3).

### 2.7. Obtaining Outputs

**Figure 6:**
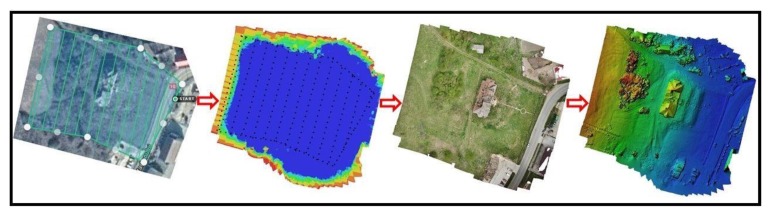
Photogrammetry processing stages.

**Figure 7:**
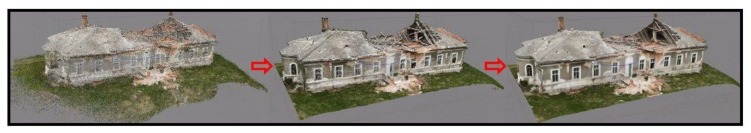
3D model reconstruction from early stages of point cloud.

To obtain the orthophoto and DEM, all suitable images were processed using Agisoft Metashape, in an established workflow that includes image alignment, followed by manual georeferencing to identify the GCPs, GNSS coordinate assignment to the matched pictures, optimization procedure, sparse and dense point cloud generation. The framework of 3D model consisted of similar steps, starting with images acquired manually, followed by a pre-selection to remove blurred photos, image rectification, point cloud segmentation and 3D reconstruction (Figure 6). Taking into account the laws of multiple-view geometry, images of the desired model should be collected from all possible viewing directions, in order to reconstruct its accurate 3D shape and requires a camera network that possess nadir and oblique images with various baselines [23,24,25,26]. Combining the mentioned steps, adequate obtained data and the specialized software with processing power, it is viable to obtain a 3D point cloud of the heritage building and environment within a few centimeters accuracy (Figure 7).

**Figure 8:**
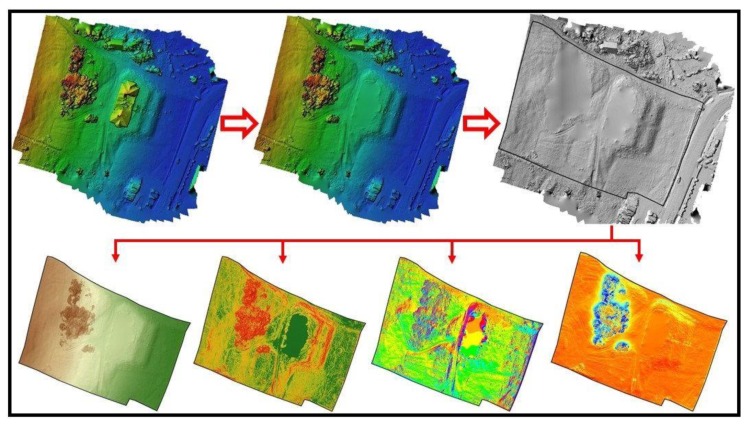
Spatial analysis stages of the surrounding heritage area.

A spatial analysis of the heritage buildings’ surrounding was performed using the centimeter resolution digital elevation model obtained using UAV technology and the previous mentioned stages. Prior to the spatial analysis, a digital surface model was created by removing the mansion footprint from the point dense cloud and interpolating the rest of the surface. In this way, the construction will not affect the spatial analysis and the elaborated figures consisting of digital surface model (DSM), slope, aspect, solar radiation will be more reliable as well as the interpretation of the results (Figure 8).

**Figure 9:**
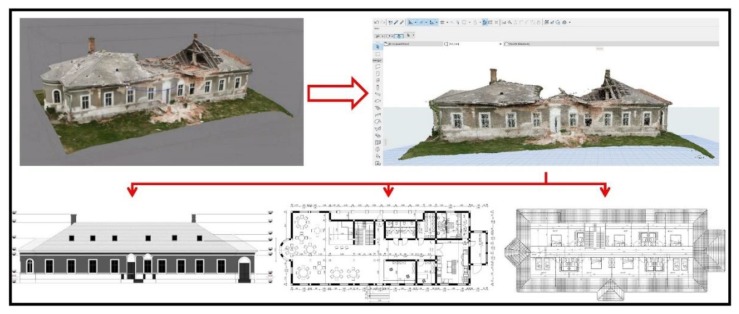
SfM 3D model export in architectural software.

After the acquisition of the field data and the creation of the complex 3D model necessary in architectural projects, it is necessary to deliver reliable outputs consisting of a 3D database with spatial and metric information that can be used by civil engineering, architects, land surveyors and contractors to exchange crucial information to improve the productivity and efficiency in complex restoration projects and to aid the construction process. Such study would incorporate detailed and accurate collected measurements, with smart integration of exported UAV data into the Building Information Modelling (BIM) software [27,28]. A preliminary prospect in order to establish the feasibility of such rehabilitation of a heritage building and the proposed transformation into a touristic pension and restaurant was achieved using ArchiCAD software to recreate a facade and architectural plans for the ground floor and finished attic (Figure 9).

## 3. Results

### 3.1. Orthophoto and 3D Model 

This current case study highlights the potential and advantages of Unmanned Aerial Vehicle (UAV) implementations in various projects regarding cultural heritage, architecture and construction in general, accompanied by various phases of spatial planning [29,30].

**Figure 10:**
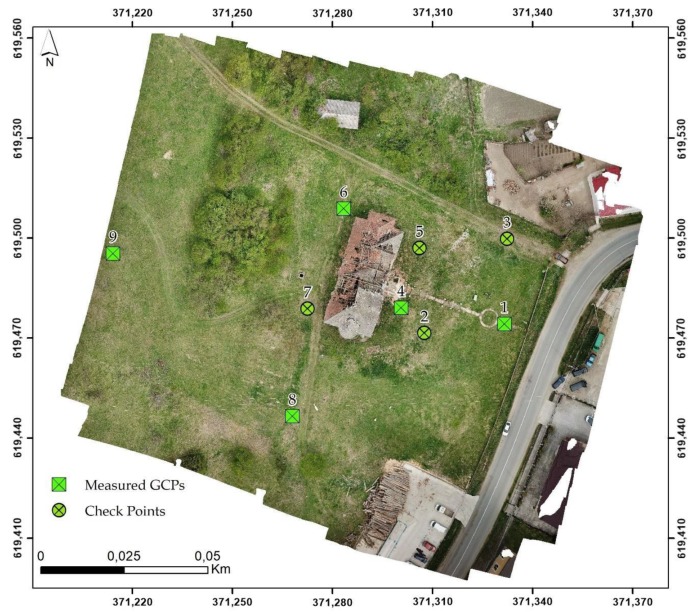
Obtained orthophoto.

The design of any construction, from old, degraded or severely damaged to new projects, cannot be carried out without up-to-date topographic plans, that require topographic and geodetic measurements obtained with specialized methods and instruments. In the current stage of industrialization of the construction process, land and construction surveys are indispensable in the activity of designing and elaborating investments such as a complex rehabilitation and restoration architectural project. It is the land surveyor’s responsibility to procure field measurement so that the terrain surface can be accurately represented, to obtain contour levels, longitudinal profiles, transversal profiles and elevations of topographical details. Topography and geodesy are based on well-established methods and instruments, they are limited to tedious tasks, time-consuming stages, prone to injuries and relatively expensive. Throughout the presented steps, the feasibility and potential of UAV obtained data, combined with specialized software and the processing power required, to obtain accurate and complex 3D surveyed models, consisting of hundreds of thousands of point clouds. The generated deliverables consist of orthophoto and DEM that have up to centimeter-level resolution (Figure 10) further used to create useful outputs in the design and construction project, such as: contour lines, longitudinal and transversal profiles, volumetric and surface calculations. Such spatial analysis can provide useful and complementary information to geotechnical investigation and help the civil engineers in the design, construction and earthworks of foundations and retaining walls.

**Figure 11:**
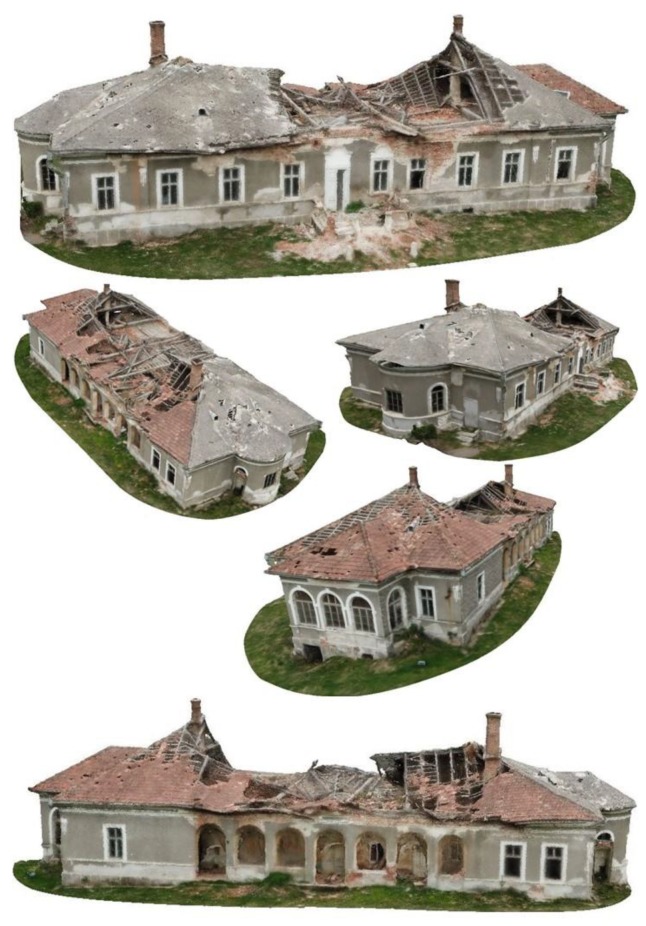
Photorealistic 3D model.

3D reconstruction through Structure from Motion (SfM) software, remote sensing technologies and UAV-obtained imagery is a powerful tool to provide accurate 3D models and point clouds essential in any desideratum to obtain a high-resolution photorealistic representation of buildings and cultural heritage sites (Figure 11). The overall quality of a result is directly proportional to the quality of the acquired images, position and GCPs used. The obtained result successfully offers excellent opportunities to further analyze the construction for damage inspections, cost estimate for restoration, assessment of conservation to the cultural heritage building, point dense cloud similar to the one obtained by terrain laser scanner and a better decision-making process. 

The final 3D reconstruction provides architects, civil engineers, land surveyors, archaeologists and local authorities with high-resolution data for rapid mapping of cultural heritage buildings [5,8,10]. The accuracy and completeness of the presented 3D model allows structural deformation analysis to also be undertaken from the comfort of office buildings in real time by interdisciplinary engineers, saving numerous on-site visits and implicitly work hours, costs and potential dangers [25].

### 3.2. Structural Analysis and Rehabilitation-Restoration Proposal

Given the significant advantages of incorporating UAV technology in heritage architecture, construction and land survey, the collected data needs to be merged with the technology and software used by the end-user, i.e., the architect, civil engineer, structural engineer, etc. Despite the absence of an interior model because of the advanced state of degradation and the real danger of building collapse, having a complete exterior model of the researched mansion facilitates further applications. Interdisciplinary projects are challenged by the need to establish a UAV-BIM framework in order to integrate sensed data and productivity software, thus creating a link between collected data and existing BIM platforms. 

**Figure 12:**
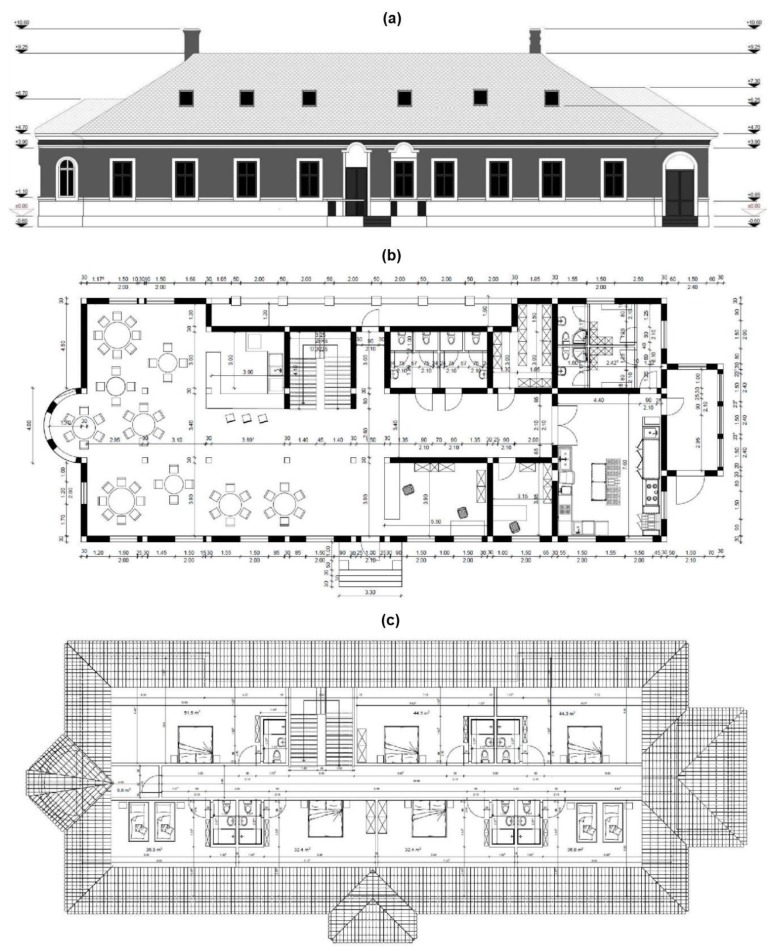
Proposed rehabilitation and restoration; (**a**) Reconstructed facade; (**b**) Envisioned ground floor; (**c**) Envisioned attic.

Using the photorealistic and accurate 3D model that incorporates useful spatial data and metrics indispensable in architecture and construction design, smart integration of exported UAV data into the BIM software (ArchiCAD) was achieved. Thus, taking into account the historic background of Hatfaludy Mansion, its configuration, the geographic location, tourism potential and presented touristic attractions, accompanied by an accessibility study further presented in the next sub chapter, a preliminary prospect for the utilization of the historic mansion was created. The feasible solution would be the transformation and arrangement into a pension and restaurant (Figure 12). Taking into account that the mansion is a heritage building, the rehabilitation and restoration must be done with regards to the Heritage Legislation and with minimum changes to the facade, height regime, construction footprint etc. [31,32]. In consequence, the envisioned prospect respects the history and the potential of the region, is intended to serve future tourists and local residents. The architectural plans consist of a ground floor conceived as a restaurant with a bar, reception, manager’s office, kitchen and utilities, and the finished attic as seven rooms for accommodation.

**Figure 13:**
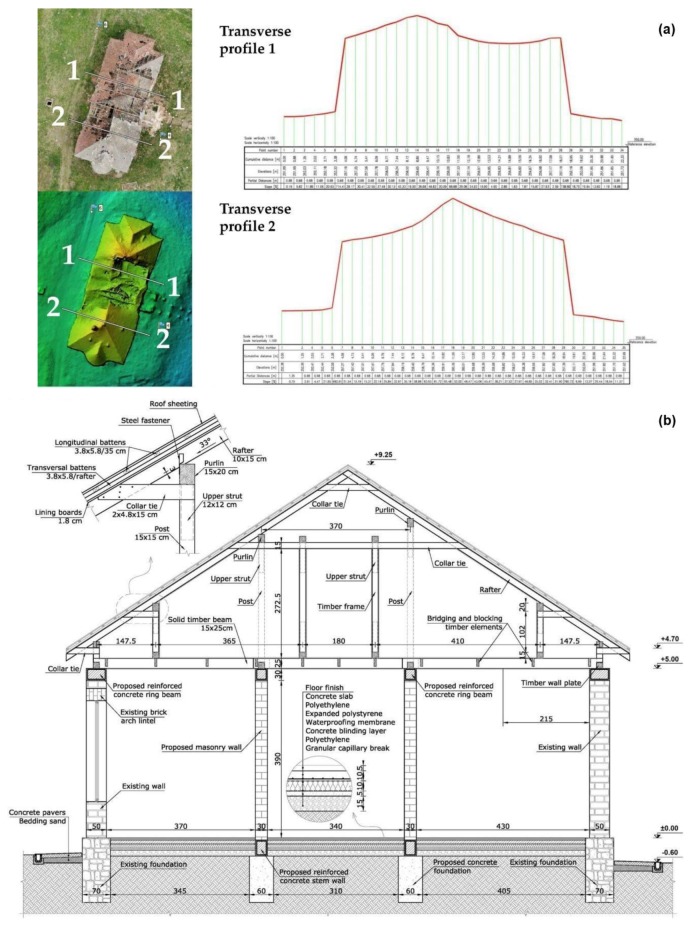
(**a**) Extracting metric data from transverse profiles; (**b**) Envisioned section.

Inspecting the obtained 3D model it can be seen that, from the structural point of view, the mansion is in an advanced state of degradation: the roof is partially collapsed, and the brickwork and timber floor structural elements are damaged due to water infiltration. The obtained UAV survey provides valuable metrics such as wall heights and dimensions, ridge elevation, roof angles, etc. necessary for structural analysis and the development of a feasibility assessment with respect to the envisioned restoration proposal. Based on the observed and measured exterior thick brick walls specific for such constructions in the 19th century, they can serve as load-bearing walls for the light wood structure. A set of primary structural interventions are mandatory. In the first phase, the remaining parts of the roof need to be demolished together with the timber floor and furthermore the exterior walls need to be propped in order to ensure their stability during the rehabilitation works. After the damaged masonry is replaced or repaired, a reinforced concrete ring beam (missing in the current state) should be erected at the top end of the walls which will tie up the whole masonry, will enhance the structural stability and will uniformize the loads transferred from the proposed lightweight timber roof and floor (Figure 13). The evaluated pressure transmitted by the foundations to the soil is about 125 kPa, calculated considering that the width of the existing foundations is 50 cm, which is below the conventional pressure associated with the soils that fall under the geographical localization of the building.

### 3.3. Spatial Analysis

The relief, through its characteristics is the main factor that conditions the location of the residential infrastructures. The main characteristics that induces restrictiveness are the slope of the terrain and the orientation of the inclined surfaces.

In contrast to the restrictiveness, the relief forms offer favorability from the point of view of enhancing any residential infrastructure. Thus, referring to the case studied and analyzing in comparison the morphometric features of relief (slope and altitude) obtained as a result of the integrated analysis of the UAV images, highlights the fact that the relief of the analyzed location has undergone anthropic changes to improve the construction developed on the terrain.

**Figure 14:**
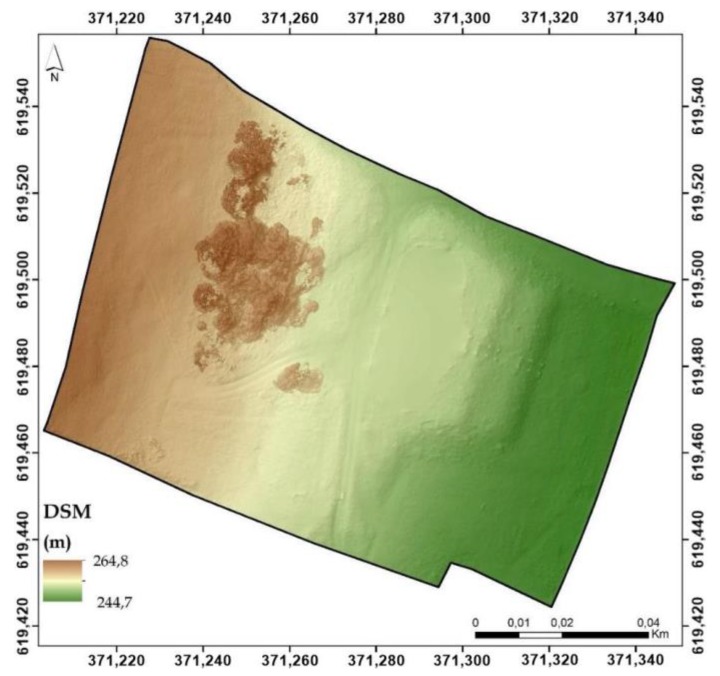
Digital surface area.

The analysis of the digital database in raster format, represented by the DSM, illustrates two terraces made anthropically (Figure 14). The first terrace located between 247 and 250 m has the role of a step between the actual construction and the arrangements in the mansion’s yard. The second terrace, also realized anthropic, is located between 250 and 252 m and has a supporting role for the construction footprint and for highlighting it as a panoramic location. The anthropic character of the morphometric terraces is highlighted by the slopes of the extremities that borders them.

**Figure 15:**
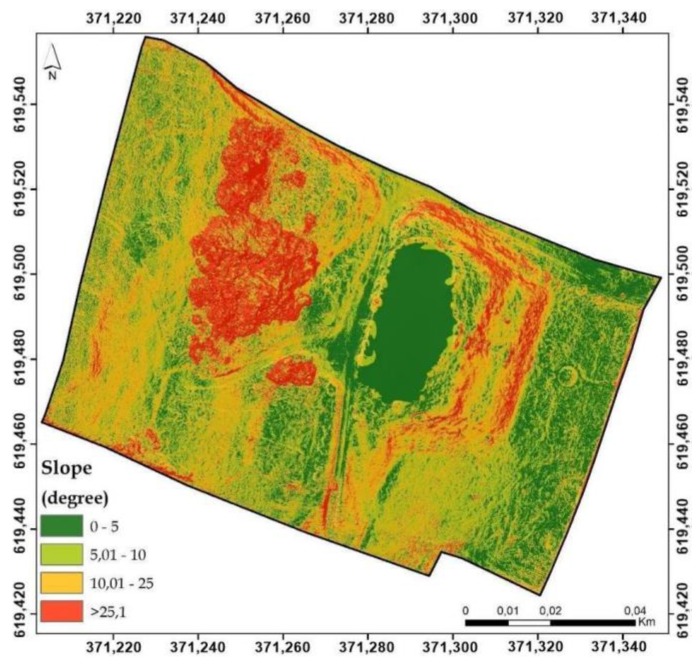
Slope illustration.

From the slopes’ point of view, the big slope of the two fronts of the terrace as well as the very high slope of the north-eastern extremity of the two terraces stand out very clearly, which highlights the earthworks previously conducted. At the same time, the small slopes modeled for the upper terrace (the terrace on which the mansion is built) located between 5.1 and 10 degrees and the first terrace with the garden of the mansion are evident, which highlights the three morphometric steps and the transition surfaces between them (Figure 15).

Also, the anthropic intervention regarding the arrangement and modification of the relief for the construction of the building is highlighted by a large slope modeled for the south-east and south slope, which materializes in the main passage between the second terrace and the southern part of the garden. At the same time the slope and the anthropic intervention is highlighted in the south-western part where large slopes are linearly modeled, between 10.1 and 25 degrees, slope that represents the step between the forestry surfaces of the upper morphometric floor behind the mansion.

**Figure 16:**
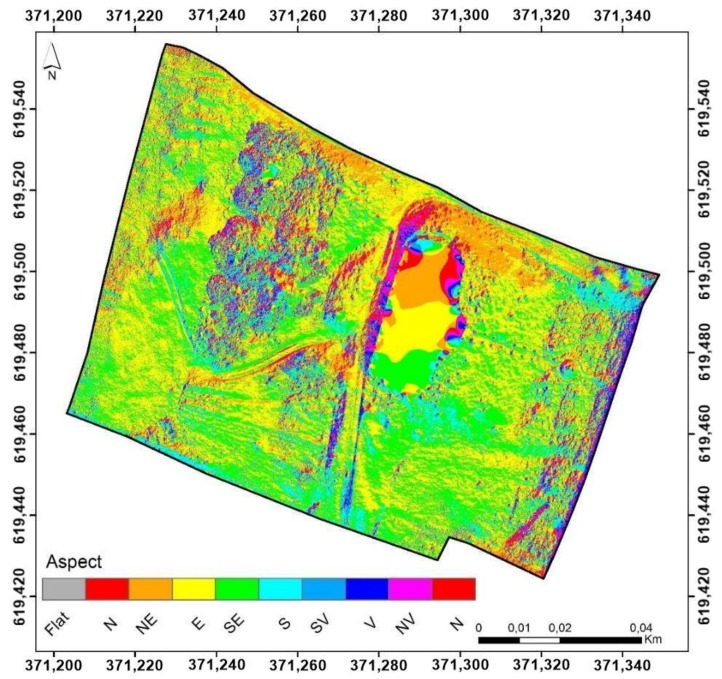
Aspect illustration.

As previously mentioned, the orientation of the inclined surfaces and implicitly the orientation of the analyzed residential infrastructure represents an important element in its exploitation during the cold season. The analysis of the raster database, modeled on the basis of the digital surface model, shows the orientation of the infrastructure in the north-east, east, south-east, south direction, with a dominant preponderance of the north-east and east oriented surfaces, which leads to the reception of higher quantities of light and implicitly of heat (Figure 16).

**Figure 17:**
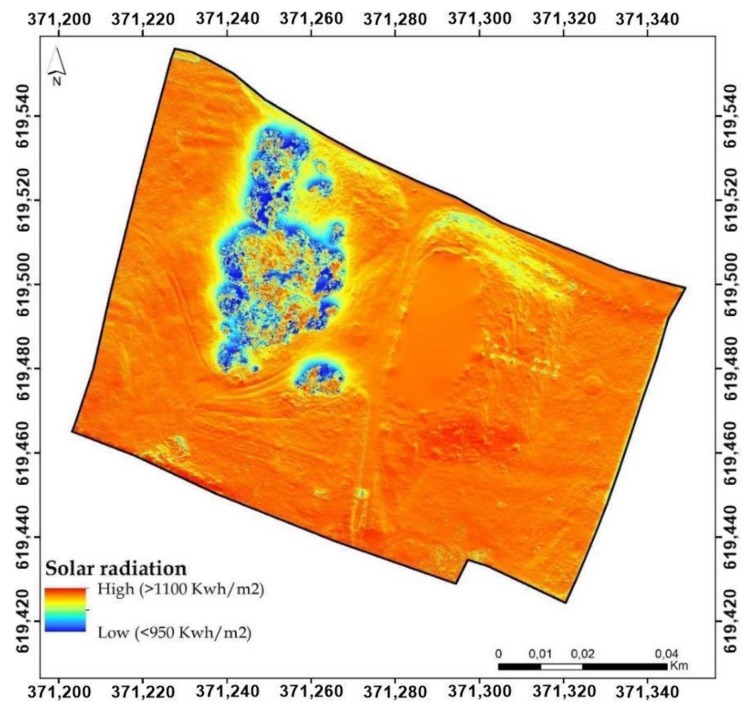
Solar radiation illustration.

Incoming solar radiation (insolation) received from the sun was modeled using solar radiation analysis tools in the ArcGIS Spatial Analyst extension. It varies depending on site position and elevation, orientation (slope and aspect), topographic characteristics, microclimate and factors such as air and soil temperature regimes, evapotranspiration, snow melt patterns, soil moisture, and light available for photosynthesis (Figure 17).

Insolation is an extremely important factor because it fuels photosynthesis in terrestrial ecosystems. At the same time, evaporation is an estimated factor by direct and indirect indices in complex inter and transdisciplinary studies. Solar radiation is one of the factors taken into account when it comes to energy efficiency of renovated or newly built constructions. This radiation reduces energy costs and increases the value and competitiveness of buildings [33,34].

The heat gain is pursued by architects by designing a larger window system on the bright sectors of buildings. Following the studies, it has been revealed that the south-facing windows transmit huge quantities of solar radiation in the winter months and small quantities in the summer months. On the other hand, specialized studies have shown that the proximity towards a forested surface reduces the amount of radiation. This fact is important for people exposed to heat islands specific to the high-density areas of buildings, also having the role of mitigating the thermal stress in hot summer conditions [34,35].

The integrated analysis of the three analyzed databases clearly illustrate the anthropic intervention regarding the modeling of the relief for the realization of the residential infrastructure by embankments and the realization of terraces for the elevation of the flat surfaces on two levels. The earthworks were most definitely conducted with the main purpose of enhancing the infrastructure, both from an architectural point of view and from a visual impact point of view, by offering an imposing aspect. At the same time, the location of the residential infrastructure on the second anthropic terrace gives the mansion a panoramic point of view of the adjacent territories, creating a pleasant panorama.

### 3.4. Tourist Infrastructure Accessibility

Accessibility is an essential condition for the development of tourist infrastructures. Highlighting the favored position as a central place compared to the main urban centers and axes of tourist flows can be highlighted based on the temporal accessibility of the surfaces adjacent to the analyzed objective [36,37,38,39,40]. For this purpose, a spatial analysis model was developed based on vector format data (roads, urban areas), attribute (average travel speeds on road sectors, road indications, names of localities) and raster (travel speeds on categories of road and pedestrian) (Table 4). 

**Figure 18:**
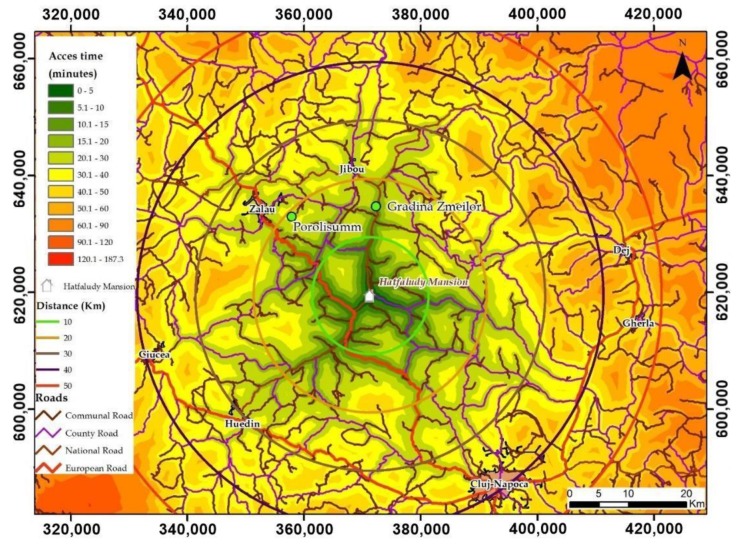
Accessibility assessment from the main surrounding cities (Cluj-Napoca and Zalau) and to the touristic locations.

All of these digital databases have been integrated into a spatial analysis model based on conversion functions, raster overlay and functions embedded in geoinformation programs to obtain a modeled raster database representing cells with access time values, measured in minutes (Figure 18).

The model of spatial analysis is based on the raster analysis of accessibility having as a starting database the raster representing travel speed/cell, raster obtained based on the calculation formulas presented by Juliao [40], Drobne et al. [38,39] and implemented in the Romanian studies by Bilasco et al. [36,37]. The presented equation was obtained using the Raster Calculator of the ArcGIS geo-informational software:

(1) $$CCT=\frac{PS*60}{TS*1000},$$

where: CCT—cell crossing time (minutes), PS—pixel size (4 m, in this case) and TS—Travel Speed (obtained as a result of the conversion of the vector file representing the roads in raster format based on the attribute column representing the speed of movement on the road sectors; for the territorial areas that are not served by roads, the average travel speed of 4 km/h was chosen, represented by the average speed of movement of a person in pedestrian regime).

The development of the Hatfaludy Mansion leads to the development of Hida Village by attracting tourist flows both from the point of view of classical tourism (simple sightseeing of a touristic object) and from the point of view of the related cross services: bed, meal and recreation. The central position of the Hida Village, related to the main axes of the flow of tourists (Cluj-Napoca-Huedin-Ciucea axis, Cluj-Napoca-Gherla-Dej axis, Dej-Jibou-Zalău axis, Zalău-Ciucea axis and Cluj-Napoca-Zalău axis) is highlighted both by the very high degree of accessibility that it registers in relation to these axes and in terms of the accessibility/time/distance ratio.

The access times related to the big urban centers that represent the ends of the main axis (Cluj-Napoca-Zalău) that transit through the cities, urban centers which are county capital cities fall in the category of “20–40 min”, representing a small travel time which represents a high attractiveness factor for the population of these cities. The rest of the axes ends (Dej, Zalău, Ciucea, Huedin, Cluj-Napoca) which border areas with maximum access times that fall into the category up to a maximum of 40 min, highlight the easy access of tourists from any direction or any route which would have been contoured at a distance of about 45 km, thus making it possible to deviate from the initial route for both sight-seeing, meal serving or accommodation, at Hatfaludy Mansion.

Approaching both in terms of travel time (30–40 min) and in terms of distance (about 20 km), the main tourist objectives of Sălaj County represented by the Roman settlement from Porolissum, Jibou Botanical Garden, Strâmba Monastery, Dragons’ Garden and many more (15–20 min and about 20 km), highlights the favorable and sustainable development in terms of attracting a large number of tourists, the development of the local community and the adjacent infrastructures.

The accessibility analysis based on the access times to the observed objective, clearly highlights the opportunity of its development in order to maximize profits and to highlight the specific architecture in order to feasibly rehabilitate, restore and transform the degrading mansion into a pension and restaurant and further include this heritage building in the main touristic routes for visiting and accommodation with provided meal servings and touristic activities.

## 4. Discussions and Conclusions

Constructed 3D models, project outputs and research steps can be employed in a great number of historical buildings that are in a degraded state or seriously damaged. The accurate survey of the architecture and its structure is advisable to different applications in the future, such as Building Information Modelling (BIM) and generating in a short period valid mapping of cultural heritage domain as well as in general, while ensuring acceptable quality [2,5,9].

The use of UAVs in interdisciplinary applications including cultural heritage buildings and constructions in general, historic sites, archaeological areas, land monitoring and survey has showed increasing interest in last decade [41,42]. Drones have become a preferred instrument for documentation, surveys, modelling, mapping, inspections and various monitoring purposes. Even though the initial cost of a UAV system can be high, the amortization period is relatively small. Surveyors can drastically increase their productivity, being able to carry out more projects in similar amount of time; drones present no safety risk for the users and eliminates injuries and ground risks. Drone solutions have become a strong alternative for geodetic surveys, ground surveys in dangerous and inaccessible places, which makes it possible to conduct better, more thorough planning [1,2,22].

An elaborate feasibility assessment regarding the potential for using UAV photogrammetry in 3D digital documentation of cultural heritage sites, presentation of various outputs and deliverables based on the obtained data to be used by interdisciplinary specialists and engineers, integration in BIM platforms for future prospect design, together with a spatial analysis using the centimeter-resolution orthoimage and DEM, accessibility and touristic potential analysis, demonstrate the promising results achieved [8,9].

By combining the presented UAV survey, structural analysis and rehabilitation proposal elements, touristic potential analysis and accessibility study in this established framework, we obtain a complete feasibility solution that is crucial in many sectors. Due to the large number of degraded and in poor state cultural heritage buildings and touristic objectives in Romania and East Europe in general, the workflow can be used as a SWAT analysis by the public and private sector in the decision making process. Both the cultural institutions or public authorities and the private investors will be greatly advantaged when using this workflow in order to highlight and identify the best and most valuable locations and edifices worthy of investing money and manpower. The output used in the decision making is the current state and if the building can sustain a rehabilitation procedure in accordance to the structure analysis and the heritage conservation laws, the touristic potential of the region and the accessibility to these locations.

The socio-cultural aspect plays an important role in the harmonious development of a community, region, local economy and prosperity, and is often neglected by the authorities and local administrations [43,44,45]. By producing 3D models and topographic surveys of existing cultural heritage buildings and monuments, we increase the visibility and potential of touristic areas, increase architectural knowledge, help preserve in digital format the status of buildings in an advanced state of degradation and also enable future construction rehabilitation and restoration. Such interdisciplinary research and projects should be a moral duty to preserve the cultural values of every country and an imperative objective. UAV scanning projects can greatly improve the work and efficiency of heritage conservation staff, government departments and local authorities that should work together for the noble cause of restoring architectural, cultural and heritage sites and buildings.

## Figures and Tables

**Table 1:** UAV and ground survey details.

	Flight Plan Properties	
Aircraft	DJI Mavic Pro	
Weight	743 g	
Date	12th May 2019	
Mapping Flight Speed	3 m/s	
Flight Time	~20 min	
Sensor	FC220_4.7_4000 × 3000 (RGB) f/2.2	
Takeoff Height Ground Level (m)	247.5 m	
Fly height ground level (m)	35 m	
Image Forward Overlap (%)	85%	
Image Side Overlap (%)	80%	
Image Overlap	>9	
Number of Images Captured	312 (automated flight—Orthophoto)	232 (manual flight—3D model)
Covered Area [m2]	~21,000	
Number of GCPs	9	
Ground Resolution	~1.06 cm/px	

**Table 2:** UAV-SfM model accuracy assessment GCPs.

GCP	Field Survey Data			Deviation		
	X (m)	Y (m)	Z (m)	dX (m)	dY (m)	dZ (m)
1	619474.228	371331.618	245.206	0.0278	−0.0131	0.0374
4	619479.051	371300.619	251.443	−0.0142	0.0311	0.0272
6	619508.855	371283.426	251.380	0.0222	0.0339	0.0574
8	619446.571	371268.081	252.540	−0.0292	−0.0262	0.0482
9	619495.296	371214.233	262.309	0.0127	−0.0186	−0.0215
	**RMSE H = sqrt ((sum(dX)^2^ + (sum(dY)^2^)/n)**			**0.034 m**		
	**RMSE V= sqrt (sum(dZ)^2^/n)**			**0.041 m**		

**Table 3:** UAV-SfM model accuracy assessment CPs.

CP	Field Survey Data			Deviation		
	X (m)	Y (m)	Z (m)	dX (m)	dY (m)	dZ (m)
2	619471.452	371307.635	248.807	−0.0493	0.0093	0.0389
3	619499.665	371332.425	245.582	−0.0196	0.0138	−0.0292
5	619496.966	371306.138	251.201	0.0104	0.0424	0.0390
7	619478.691	371272.559	252.528	0.0028	0.0193	0.0552
	**RMSE H = sqrt ((sum(dX)^2^ + (sum(dY)^2^)/n)**			**0.036 m**		
	**RMSE V= sqrt (sum(dZ)^2^/n)**			**0.042 m**		

**Table 4:** Accessibility database structure.

Database Name	Structure	Type	Attributes
Hatfaludy Mansion	point	vector	X, Y coordinates, name
Communication infrastructure (roads)	line	vector	Road type, speed
Travel time speed	grid	raster	Minutes/m^2^
Accessibility	grid	raster	Minutes
Distance	polygon	vector	Distance (km)
Touristic hotspots	point	vector	X, Y coordinates, name
